# A New Strategy to Regulate the Selectivity of Photo-Mediated Catalytic Reaction

**DOI:** 10.3389/fchem.2021.673857

**Published:** 2021-08-09

**Authors:** Kun Qian

**Affiliations:** Hefei National Laboratory for Physical Sciences at the Microscale, CAS Key Laboratory of Materials for Energy Conversion and Department of Chemical Physics, University of Science and Technology of China, Hefei, China

**Keywords:** selectivity, photo-mediated charge, reaction pathway, timescale of reaction, quantum efficiency

## Abstract

Here we developed a new method for regulating the selectivity of photo-mediated catalytic reaction by manipulating the surface charge of Au/TiO_2_ (gold/titanium dioxide) catalysts within chemical reaction timescales. Two kinds of photocatalytic reactions, hydrogenation of acetophenone and benzyl alcohol oxidation, have been applied to investigate the photocatalytic performance over Au/TiO_2_ catalysts with tunable surface charges. We found that a suitable timescale of switching surface charge on Au would benefit for the enhanced quantum efficiency and play different roles in the selectivity of desired products in hydrogenation and oxidation reactions. Au/TiO_2_ catalyst under 5 μs flashing light irradiation exhibits much higher selectivity of 1-phenylethanol in the hydrogenation of acetophenone than that under continuous light and 5 s flashing light irradiation; by contrast, Au/TiO_2_ catalysts under both flashing light and continuous light irradiation exhibit a similar selectivity of benzaldehyde in benzyl alcohol oxidation. Our findings will benefit for a better understanding of electronic structure–mediated reaction mechanism and be helpful for achieving highly efficient photocatalytic systems.

## Introduction

**FIGURE 1:**
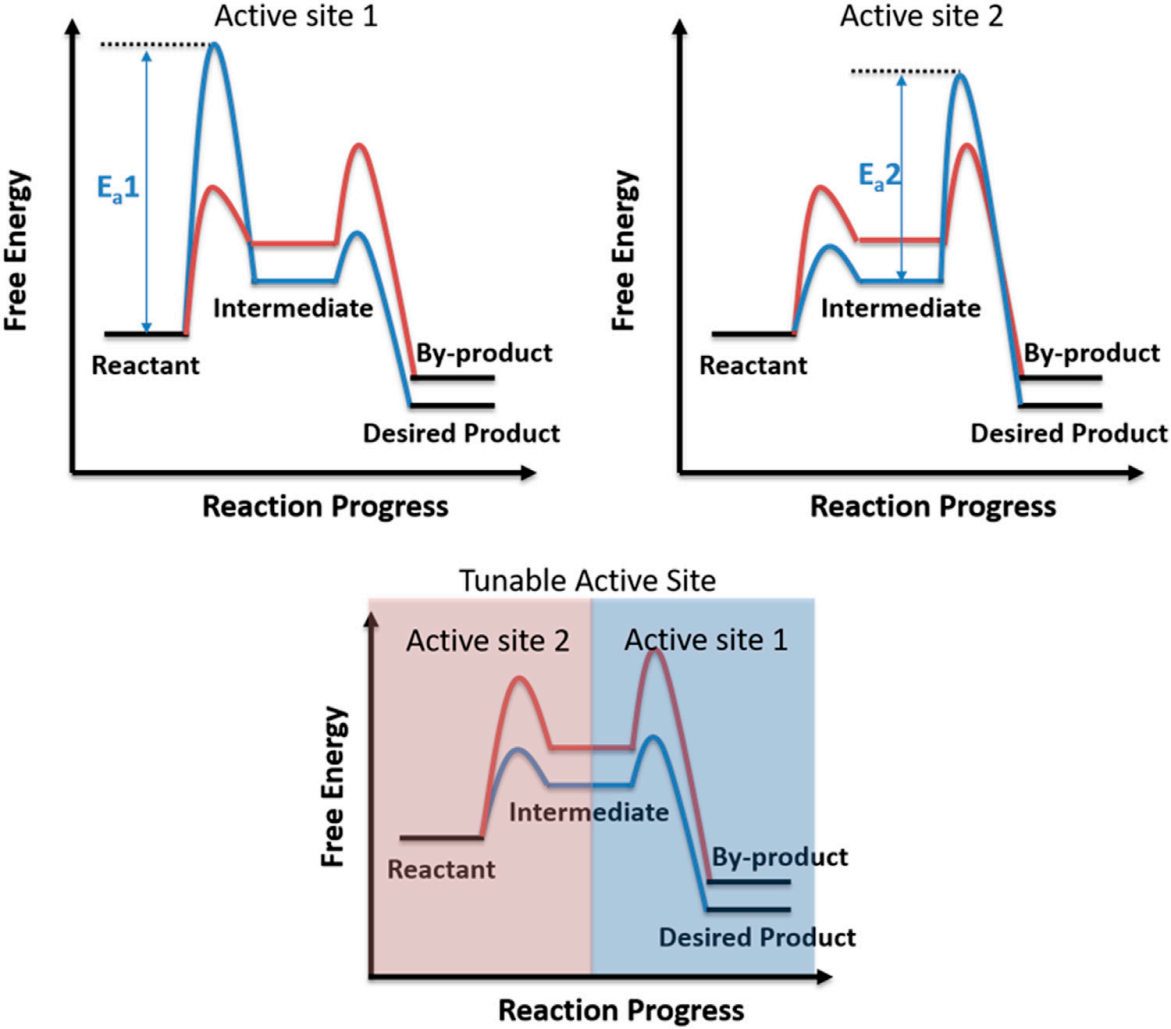
Schematic diagram of catalytic reaction pathways over metal with the tunable active site within a chemical reaction timescale.

Selectivity is one of the most important criteria for evaluating a catalytic system (Lee et al., 2009; Somorjai and Park 2008). The design and development of highly selective catalytic system will benefit for a green and cost-efficient catalytic application, because the reaction with higher selectivity generates fewer toxic by-products, benefits for the further separation and application of products, and utilizes the reactants more efficiently (Zaera 2009). In recent years, scientists have made great efforts into developing highly selective catalytic system, and several successful examples have been achieved. The concepts of single-atom catalysis and single-site catalysis have been developing rapidly in the past 10 years and now have been widely accepted and considered as highly effective catalysts for increasing the selectivity of desired products (Li et al., 2020; Wang et al., 2018; Yang et al., 2013). In the last 5 years, Chinese researchers have made a huge breakthrough in the selective conversion of syngas to light olefins by applying bifunctional catalysts, in which the zeolites would convert reaction intermediates generated from the oxides and totally change the product thermodynamic distributions (Cheng et al., 2016; Jiao et al., 2016). Wang’s group further introduced a concept of relay catalysis and achieved high-yield C_2+_ products based on these findings (Zhou et al., 2018). Similar to the bifunctional catalysts, an emerging approach named chemical looping partial oxidation (CLPO) has been developed and successfully applied to achieve near 100% CO selectivity for methane utilization, in which the redox cycle takes place in an interconnected reactor with a reducer and an oxidizer (Zeng et al., 2018; Liu et al., 2019). Until now, most successful attempts of highly selective systems are based on the optimization of catalytic active sites or the modulation of thermodynamic equilibrium, in which suitable and uniform active sites with lower energy barrier toward the designed pathway would benefit for achieving highly selective desired products, and the concept of “tandem catalysis” and “chemical looping” would optimize the product thermodynamic distributions. Notably, there are multi-steps or multi-pathways in one catalytic reaction, and the “suitable” active site might be only suitable for the rate-determining step rather than being suitable for all the steps. The design of the tunable active site within a chemical step timescale might be a good strategy to optimize the performance of catalytic reaction. More specifically, as shown in Figure 1, both active site 1 and active site 2 are not favorable to selectively form the desired product since the heights of energy barriers (E_a_1 and E_a_2) in the formation of desired products are much larger than those in the formation of by-products. By contrast, the tunable active site within a chemical reaction timescale would build up a new reaction pathway and greatly enhance the selectivity of the desired product. However, most chemical reaction timescales are within the range from μs to ms; it is almost impossible to manipulate the active site in such a short timescale.

Recently, we developed a new method for manipulating the surface charges of photocatalyst within chemical reaction timescales by applying a home-modified LED light source (Qian and Huang 2019), in which the electronic structures of supported metal NPs could be tunable within the timescale from s to μs. These findings provide us a great opportunity to design a reaction channel like chemical loop by alternately controlling the electronic structures of metal NPs within the timescale of an elementary reaction, which might be helpful to design a highly active catalytic system. In this study, we investigated the catalytic performance of metal with tunable charge by applying both hydrogenation of acetophenone and benzyl alcohol oxidation over Au/TiO_2_ photocatalysts. Similar to our previous findings, a suitable timescale of switching surface charge on Au would benefit for the enhanced quantum efficiency in both hydrogenation of acetophenone and benzyl alcohol oxidation. Interestingly, Au/TiO_2_ catalyst under 5 μs flashing light irradiation exhibits much higher selectivity of 1-phenylethanol in the hydrogenation of acetophenone than Au/TiO_2_ catalyst under continuous light and 5 s flashing light irradiation; by contrast, Au/TiO_2_ catalysts under both flashing light and continuous light irradiation exhibit a similar selectivity of benzaldehyde in benzyl alcohol oxidation. Our results provide a new method for regulating the selectivity of photo-mediated catalytic reaction by manipulating the surface charge of supported metal NPs, which would be helpful for further achieving highly efficient photocatalytic systems.

## Experimental Section

**Photocatalyst synthesis**: Au/TiO_2_ heterostructure with 3% weight percentages (3%) of Au on TiO_2_ (anatase) was prepared using deposition precipitation (DP) method (Qian et al., 2013; Qian et al., 2018). During the DP procedure, HAuCl_4_•3H_2_O (≥99.9%, Aldrich) was dissolved in Nanopure water to prepare a 0.020 M aqueous solution of HAuCl_4_. The aqueous solution of HAuCl_4_, TiO_2_ (≥99.5% trace metals basis, Aldrich), and distilled water was added to a flask and stirred vigorously at 323 K for 30 min. An appropriate amount of ammonium hydroxide (ACS reagent) was added to adjust the pH value of the system to ∼8, and the system was stirred at 333 K for 4 h. The solid was centrifuged, washed with Nanopure water three times, dried at 333 K for 12 h, and then calcined at 473 K for 4 h.

**Home-modified LED light source**: 365 nm LED light (Thorlabs M365LP1) was connected with an advanced four-channel LED driver (Thorlabs DC4104). The external control mode was chosen in our study, in which the LED light was controlled by square wave signals generated by a 30-MHz synthesized function generator. The irradiation time is equal to the dark period within one cycle by applying a square wave signal, and the circulating period was further controlled by changing the frequencies of generated signals. In our research, the cyclic frequency was designed from 0.1 to 1 × 10^5^ Hz, resulting that the cycle time is from 10 s to 10 μs and the irradiation time is from 5 s to 5 μs in each cycle (Qian and Huang 2019).

### Photocatalytic Reaction Test

**The hydrogenation of acetophenone** was performed following the method described in the previous report and conducted under an argon atmosphere at 30°C (Ke et al., 2012). Typically, 3 mmol acetophenone was dissolved into 30 ml isopropanol, and 3 ml solution of 0.1 M KOH in isopropanal and 50 mg of the 3% Au/TiO_2_ catalyst were added into the mixture that was then stirred by a magnetic stirrer during the reaction and illuminated by a 365-nm LED light with controllable frequencies. The light intensity was 0.28 W/cm^2^, and the irradiation area is about 6.5 cm^2^. During these reactions, 0.5 ml of the mixture were collected at a given irradiation time and filtered through centrifugation to remove the Au/TiO_2_ catalyst. The filtrates were analyzed by a gas chromatograph GC-7806 equipped with HP-5 column.

**The benzyl alcohol oxidation** was performed following the method described in the previous report and conducted under an O_2_ atmosphere at 30°C (Tanaka et al., 2012). Typically, 100 mg of the 3% Au/TiO_2_ catalyst were added into 10 ml distilled water, bubbled with O_2_, and sealed with a rubber septum. 20 μmol benzyl alcohol was injected into the suspension and then illuminated by a 365-nm LED light with controllable frequencies. The light intensity was 0.28 W/cm^2^, and the irradiation area is about 2 cm^2^. During these reactions, the amount of carbon dioxide (CO_2_) in the gas phase was measured using a gas chromatograph GC-7806 equipped with a Porapak QS column. The amounts of benzyl alcohol and benzaldehyde in the liquid phase were determined with a Shimadzu GC-7806 gas chromatograph equipped with a DB-1 capillary column. Toluene was used as an internal standard sample.

**Material characterization**: Powder X-ray diffraction (XRD) patterns were recorded in the 2θ at the range of 20°–80° on a Philips X’Pert Pro Super diffractometer with Cu Kα radiation (*λ* = 0.15406 nm) operating at 40 kV and 50 mA. Transmission electron microscopy (TEM) and high-resolution transmission electron microscopy (HRTEM) images were obtained on a JEM-2100F high-resolution transmission electron microscope.

**Quantum yield measurement** was performed following the method described in the previous report (Han et al., 2012). The intensities of incident lights were measured by Extech LT300 Light Meter. The number of incident photons of flashing light was normalized by a theoretical continuous light intensity.

$$P=\frac{c*h*n}{\lambda *t},$$

$$q_{p}=\frac{n}{t\left(s\right)},$$

$$\emptyset =\frac{2k}{q_{p}},$$

where λ is taken to be 365 nm, h is Planck’s constant (in J/s), c is the speed of light (in m/s), n is the number of photons, t is the time (in second), and q_p_ is the photon flux (number of photons per second).

## Results and Discussion

**FIGURE 2:**
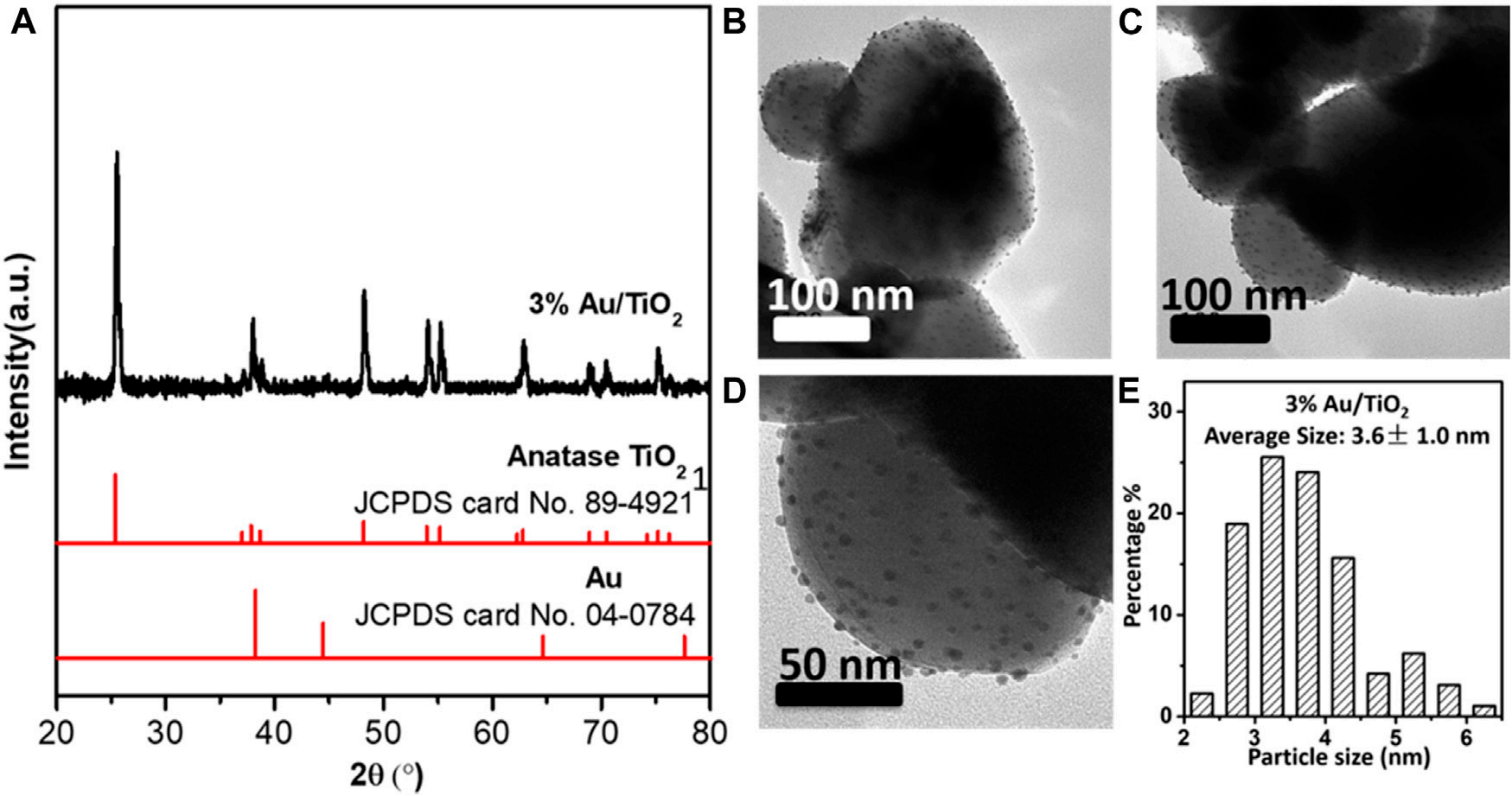
XRD **(A)**, TEM image **(B–D)**, and Au NP size distribution **(E)** of Au/TiO_2_ photocatalysts.

3%-Au/TiO_2_ catalyst was prepared by the DP method with anatase TiO_2_ as support. Figure 2A shows the X-ray diffraction (XRD) patterns of the Au/TiO_2_ catalyst. All the diffraction peaks could be assigned to the anatase TiO_2_ (JCPDS card no. 89–4,921), and no diffraction peaks attributing to Au could be identified, which suggests that the supported Au NPs should be dispersed on the TiO_2_ support (Zhou et al., 2020). Figure 2B displays the transmission electron microscopy (TEM) images and size distribution of supported Au NPs. More than 150 Au NPs have been counted for the size distribution calculation, and the average particle size of 3% Au/TiO_2_ is 3.6 ± 1.0 nm (Figure 2C).

**FIGURE 3:**
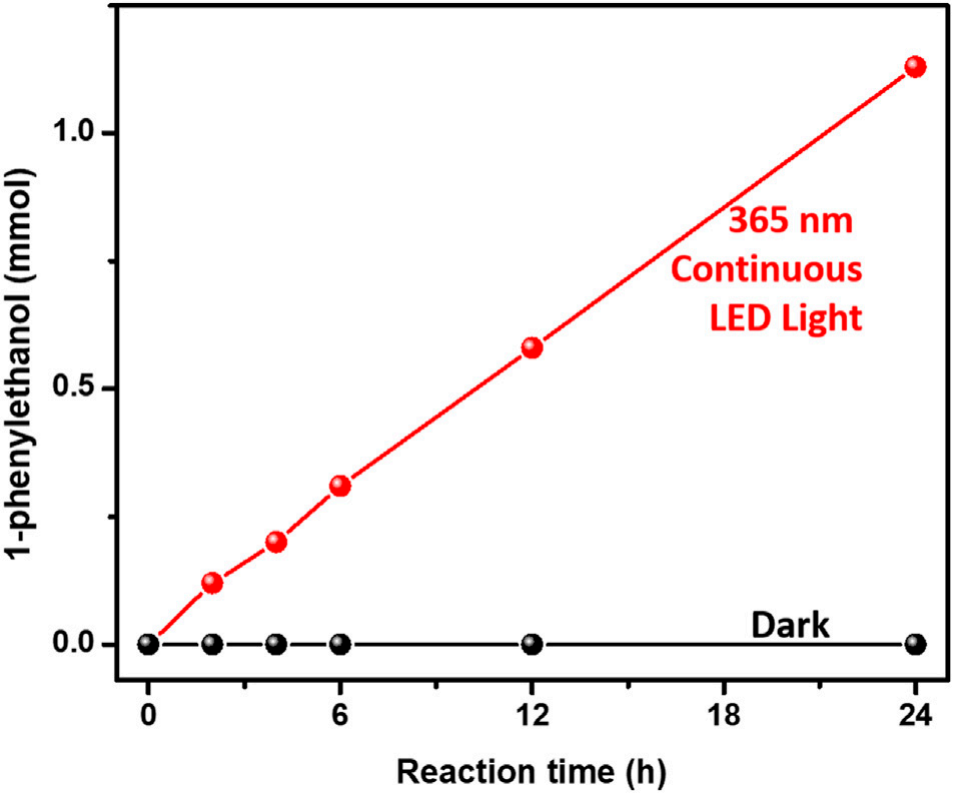
Hydrogenation of acetophenone activities over Au/TiO_2_ under and without 365 nm continuous light irradiations.

**FIGURE 4:**
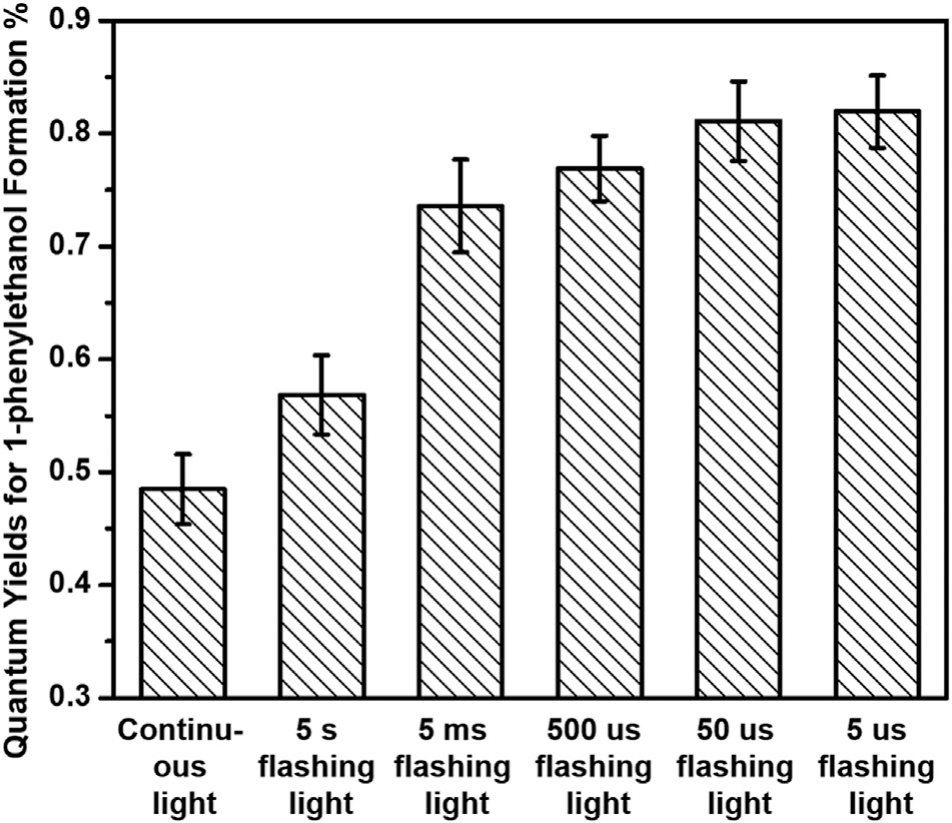
Quantum yields of 1-phenylethanol formation of hydrogenation of acetophenone over 3%-Au/TiO_2_ catalysts under flashing light with different cyclic frequency irradiations.

We further separately investigated the photocatalytic performances of both hydrogenation of acetophenone and benzyl alcohol oxidation over 3%-Au/TiO_2_ catalysts by applying our home-modified LED light with desirable irradiation times. The hydrogenation of acetophenone was conducted under an argon atmosphere at 30°C by following the method described in the previous report (Ke et al., 2012). No detectable 1-phenylethanol has been found over Au/TiO_2_ catalyst without light irradiation, suggesting that supported Au NPs could not catalyze hydrogenation of acetophenone to 1-phenylethanol. As shown in Figure 3, the amount of 1-phenylethanol production increases linearly under 365 nm continuous LED light irradiation, indicating that the reaction rate of 1-phenylethanol production does not change within our experimental conditions. The catalytic performances of hydrogenation of acetophenone over 3%-Au/TiO_2_ catalyst have also been conducted under our home-designed flashing light irradiation for 24 h, all the catalysts display a linear relationship between the time and the amount of 1-phenylethanol production, and the reaction rates of 1-phenylethanol production are 0.97 ± 0.06, 0.57 ± 0.03, 0.73 ± 0.04, 0.77 ± 0.03, 0.81 ± 0.03, and 0.82 ± 0.03 mmol×g^−1^_cat_×h^−1^ under continuous light, 5 s flashing light, 5 ms flashing light, 500 μs flashing light, 50 μs flashing light, and 5 μs flashing light, respectively. We further measured the intensities of incident lights and calculated the quantum efficiencies by following the method described in the previous reports. The quantum yields of 1-phenylethanol production at 365 nm are 0.48 ± 0.03%, 0.57 ± 0.04%, 0.74 ± 0.04%, 0.77 ± 0.03%, 0.81 ± 0.04%, and 0.82 ± 0.03% under continuous light, 5 s flashing light, 5 ms flashing light, 500 μs flashing light, 50 μs flashing light, and 5 μs flashing light, respectively, as shown in Figure 4. Similar to our previous findings in the hydrogen evolution, the quantum yields of 1-phenylethanol production under flashing lights are higher than those under continuous light. Specifically, 5 s flashing light displays a similar quantum yield with continuous light, and the quantum yield gradually increases with decreasing the irradiation time in each cycle of flashing light. 5 μs flashing light irradiation exhibits the highest quantum yield among all the flashing lights irradiations, which is 1.7 times as high as that under continuous light. These findings suggest that the quantum efficiency of photons in the hydrogenation of acetophenone has been successfully promoted by controlling photo-generated charge carriers in the timescale of hydrogenation reaction.

**FIGURE 5:**
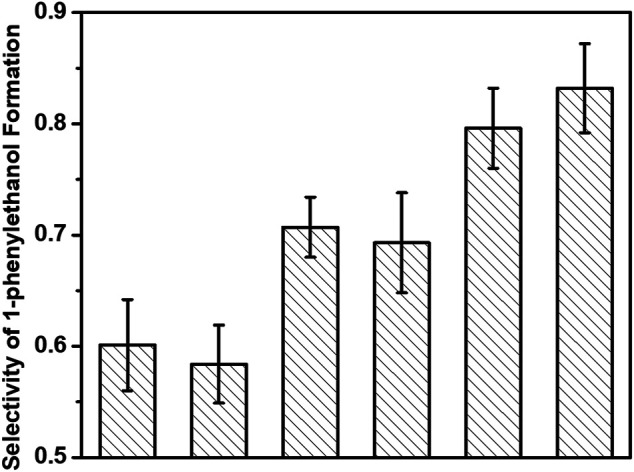
Selectivities of 1-phenylethanol formation of hydrogenation of acetophenone over 3%-Au/TiO_2_ catalysts under flashing light with different cyclic frequency irradiations.

More interestingly, we observed an increment of the selectivity of 1-phenylethanol formation with decreasing the irradiation time in each cycle of flashing light. As shown in Figure 5, the selectivities of 1-phenylethanol production in hydrogenation of acetophenone are 0.60 ± 0.04, 0.59 ± 0.04, 0.71 ± 0.03, 0.69 ± 0.05, 0.80 ± 0.04, and 0.83 ± 0.04 under continuous light, 5 s flashing light, 5 ms flashing light, 500 μs flashing light, 50 μs flashing light, and 5 μs flashing light, respectively. 5 s flashing light displays similar 1-phenylethanol selectivity with continuous light; by contrast, the 1-phenylethanol selectivity over 3%-Au/TiO_2_ catalyst under 5 μs flashing light irradiation could reach to 83%, which is much higher than those under continuous light and 5 s flashing light irradiations. Besides the quantum efficiency of photons, the selectivity of the desirable product has also been promoted by establishing a suitable timescale of switching the surface charge on supported Au with our home-modified LED irradiation.

**FIGURE 6:**
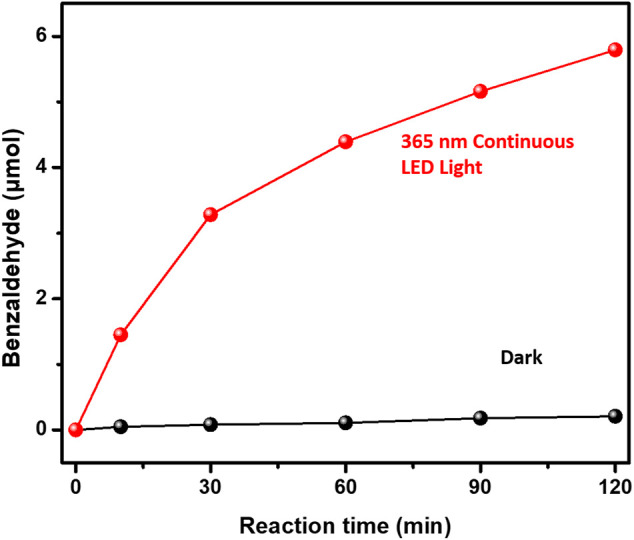
Activities of benzyl alcohol oxidation over Au/TiO_2_ under and without 365 nm continuous light irradiations.

**FIGURE 7:**
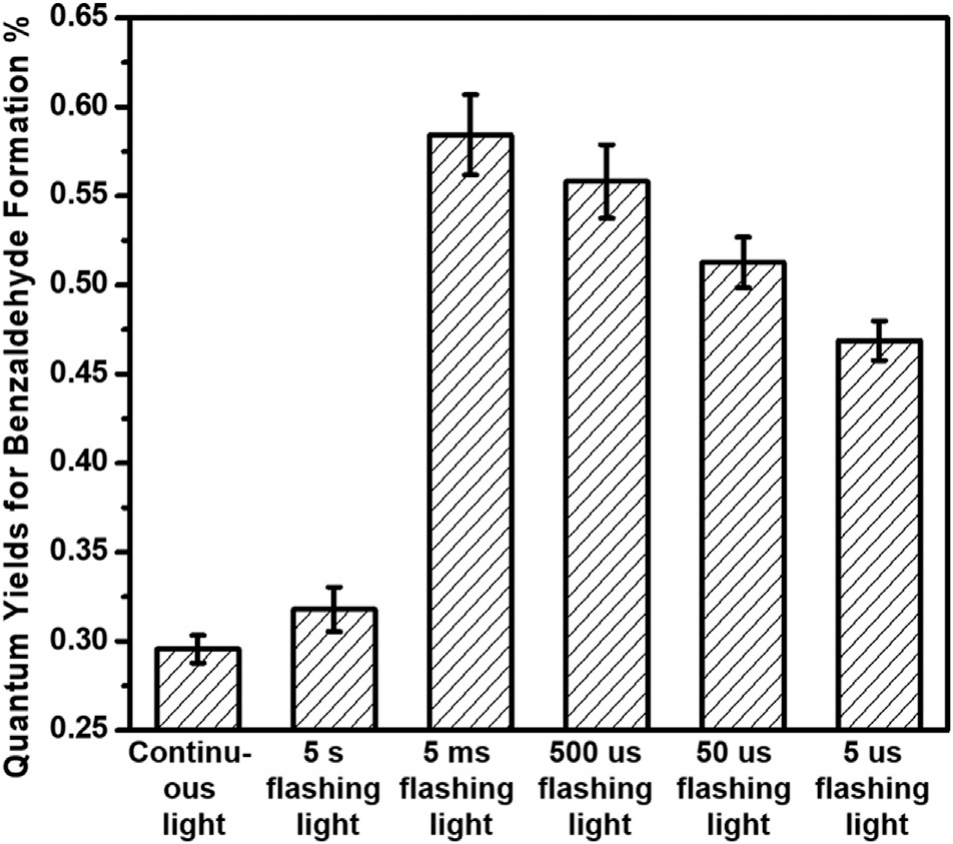
Quantum yields of benzaldehyde production of benzyl alcohol oxidation over 3%-Au/TiO_2_ catalysts under flashing light with different cyclic frequency irradiations.

The photocatalytic performances of benzyl alcohol oxidation over 3%-Au/TiO_2_ catalysts have also been investigated by following the method described in the previous report, as shown in Figure 6. Supported Au NPs could catalyze the formation of benzaldehyde without light irradiation; however, compared to the catalytic performance under the 365 nm light irradiation, the contribution from the dark reaction could be ignored. Notably, different from the linear increment of 1-phenylethanol production in the hydrogenation of acetophenone, the amount of benzaldehyde production increases nonlinearly in the benzyl alcohol oxidation, suggesting that the reaction rate of benzaldehyde production is strongly related to the concentration of the reactants, which is consistent with a previous kinetics study of benzyl alcohol oxidation (first-order reaction) (Galvanin et al., 2018). The catalytic performances under different flashing light irradiations within 120 min have also been investigated, as shown in Figure 7; the calculated reaction rates of benzaldehyde production are 91.2 ± 2.3, 48.4 ± 1.9, 89.0 ± 3.4, 85.0 ± 3.1, 78.0 ± 2.2, and 71.4 ± 1.7 μmol×g^−1^_cat_×h^−1^ under continuous light, 5 s flashing light, 5 ms flashing light, 500 μs flashing light, 50 μs flashing light, and 5 μs flashing light, respectively. We further measured the intensities of incident lights and calculated the quantum efficiencies by following the method described in the previous reports. The quantum yields of benzaldehyde production at 365 nm are 0.30 ± 0.01%, 0.32 ± 0.01%, 0.58 ± 0.02%, 0.56 ± 0.02%, 0.51 ± 0.02%, and 0.47 ± 0.01% under continuous light, 5 s flashing light, 5 ms flashing light, 500 μs flashing light, 50 μs flashing light, and 5 μs flashing light, respectively. The quantum yields of benzaldehyde production under flashing lights are higher than those under continuous light, which is consistent with our previous findings in hydrogen evolution and hydrogenation of acetophenone; 5 s flashing light displays a similar quantum yield with continuous light, while a suitable timescale 5 ms flashing light exhibits the highest quantum yield of benzaldehyde production.

**FIGURE 8:**
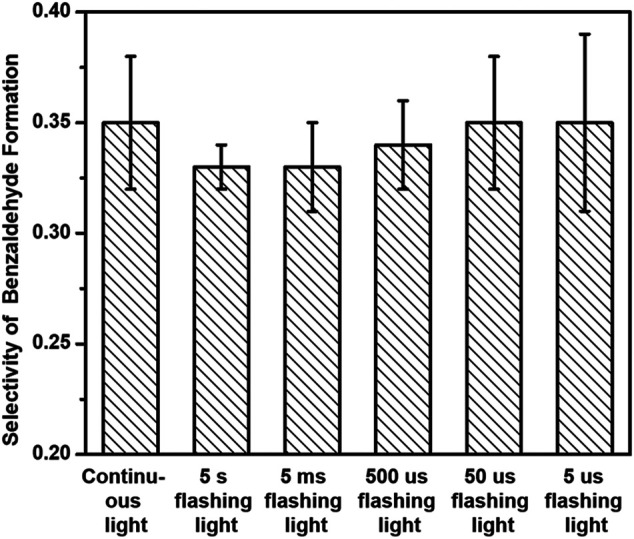
Selectivities of benzaldehyde production of benzyl alcohol oxidation over 3%-Au/TiO_2_ catalysts under flashing light with different cyclic frequency irradiations.

Figure 8 displays the selectivity of benzaldehyde production over 3%-Au/TiO_2_ catalyst with a tunable timescale of flashing light irradiation within the first half hour. The selectivities of benzaldehyde production in benzyl alcohol oxidation are 0.35 ± 0.03, 0.33 ± 0.01, 0.33 ± 0.02, 0.34 ± 0.02, 0.35 ± 0.03, and 0.35 ± 0.04 under continuous light, 5 s flashing light, 5 ms flashing light, 500 μs flashing light, 50 μs flashing light, and 5 μs flashing light, respectively. Different from the increment of the selectivity of 1-phenylethanol formation with decreasing the timescale of flashing light, Au/TiO_2_ catalysts under different flashing light irradiations exhibit a similar benzaldehyde selectivity in the benzyl alcohol oxidation, which suggests that the influence of surface electronic structure of supported Au on the benzyl alcohol oxidation should be different from that on the hydrogenation of acetophenone.

Our results demonstrate that a suitable timescale of modifying surface charges on supported Au would enhance the quantum efficiency of both hydrogenation and oxidation reactions but play different roles to the selectivity of desired products. We believe that the transferred electrons from TiO_2_ to Au with suitable lifetime contribute to this phenomenon, in which negative-charge Au, partial negative–charge Au, and neutral-charge Au have been established within the timescale from s to μs. Supported Au displays negative charge due to the existence of transferred electrons from TiO_2_ under the 365-nm continuous light irradiation, and exhibits an alternate appearance of negative charge, partial negative charge, and neutral charge with increasing the time of dark period in the flashing light. In the hydrogenation of acetophenone, the rate-determining step has been ascribed to the electron transfer process from catalyst to adsorbed acetophenone (Kohtani et al., 2012), and over-reduction will cause the formation of by-products like ethylbenzene, acetylcyclohexane, and 1-cyclohexylethanol (Fujita et al., 2016). In a cycle of our flashing light, the light irradiation period is similar to the continuous light irradiation, while the dark period with partial negative–charge and neutral-charge Au contributes to the enhanced quantum efficiency and promoted selectivity of 1-phenylethanol production. In particular, the transferred electrons from TiO_2_ to Au with suitable lifetime could directly catalyze hydrogenation of acetophenone in the dark period. Compared with the negative-charge Au under the 365-nm light irradiation, this partial negative–charge and neutral-charge Au represents low-density electrons and could prevent acetophenone from over-reduction. This is the reason that both the quantum efficiency and selectivity of 1-phenylethanol production obviously increase under our home-modified LED flashing light irradiation. In the benzyl alcohol oxidation, the photo-generated charge carriers would facilitate the formation of nonselective ˙OH radicals, which would cause an over-oxidation of benzyl alcohol and play negative role to selectively catalyze benzyl alcohol into benzaldehyde (Feng et al., 2011). Although the charge carriers within supported Au and TiO_2_ could facilitate the formation of ˙OH radicals in the dark period and increase the quantum yields of benzaldehyde production, different from the hydrogenation of acetophenone, the partial negative–charge and neutral-charge Au could only involve into the formation of ˙OH radicals rather than directly catalyzing benzyl alcohol oxidation. That is the reason we did not observe any change in the selectivity of benzaldehyde production.

## Conclusion

By investigating the photocatalytic performance over supported Au under our home-modified flashing light irradiations, we developed a new strategy to control the reaction pathway by manipulating the electronic structures of supported Au within the reaction timescale. In particular, both the quantum efficiency and selectivity of 1-phenylethanol production in the hydrogenation of acetophenone over supported Au obviously increased under flashing light irradiations, while the selectivity of benzaldehyde production in the benzyl alcohol oxidation did not change under flashing light irradiations. We believe that these findings will benefit for a better understanding of electronic structure–mediated reaction mechanism and be helpful for achieving highly efficient photocatalytic systems.

## Data Availability

The original contributions presented in the study are included in the article/Supplementary Material; further inquiries can be directed to the corresponding author.
